# Gastric Perforation after Chicken Bone Ingestion

**DOI:** 10.1155/2019/2789031

**Published:** 2019-05-15

**Authors:** Kasey Radicic, J. Thomas Dorsey, Rick Greco

**Affiliations:** ^1^Internal Medicine Resident Physician, PGY2, Ohio Valley Medical Center, Wheeling, WV, USA; ^2^Gastroenterologist, Ohio Valley Medical Center, Wheeling, WV, USA; ^3^Program Director, Internal Medicine Residency Program, Ohio Valley Medical Center, Wheeling, WV, USA

## Abstract

Chicken bone ingestion is a common occurrence that usually passes uneventfully through the digestive tract and rarely results in complications such as perforation, which occurs very rarely within the stomach. We report here a case of a chicken bone ingestion resulting in gastric perforation, which ultimately required surgical correction.

## 1. Introduction

Ingested foreign bodies (IFB) are commonly reported in mentally handicapped adults, elderly patients with dentures, and prisoners after intentional ingestion [1, 2]. IFB rarely result in complications of obstruction or perforation [3]. Most IFB are spontaneously excreted from the gastrointestinal (GI) tract without complications within one week due to protective factors including the thick, muscular GI wall with surrounding omentum [1, 4]. Less than 1% of IFB result in perforation [5], which most commonly occurs at the most narrow or angulated portion of the bowel [2]. There are reports in the literature of IFB resulting in gastric perforation, most commonly with very large or sharp objects [6]. Due to the acute angulation and narrowing of the pyloric channel, gastric perforations most commonly occur within the antrum [1].

## 2. Case

**Figure 1:**
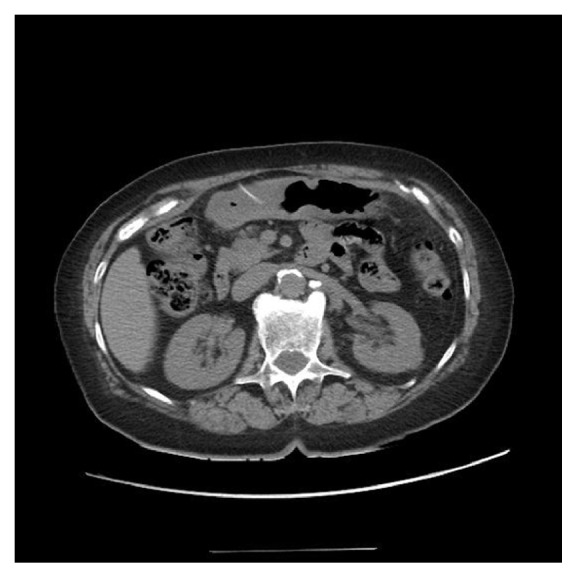
CT image of abdomen and pelvis without contrast, revealing a hyperdense, elongated, linear foreign body perforating the serosa of the stomach.

An 83-year old female with a past medical history of diverticulitis presented to the hospital with sharp, consistent, 7/10 abdominal pain that woke her up at 3 AM. The pain moved from the epigastric region to her right flank. She denied any other symptoms, including nausea/vomiting or fever/chills. The patient reported that she had eaten chicken breast at a restaurant earlier that evening. On physical exam, vital signs were stable and the abdomen exhibited diffuse tenderness. Laboratory studies revealed leukocytosis of 17.0, otherwise unremarkable. CT of the abdomen/pelvis was negative for free air/fluid collection but revealed a linear hyperdensity, approximately 2.7 cm x 2 mm, within the stomach, which penetrated through the gastric wall into the abdominal cavity (Figure 1).

The patient was informed that the CT scan may suggest an ingested foreign body, which has likely perforated through the gastric wall. Treatment options were discussed, and the patient opted to have esophagogastroduodenoscopy with attempted endoscopic removal of the foreign body. Endoscopy revealed a region of inflammation and granulation tissue of the distal antral gastric mucosa. Biopsy forceps were used to obtain biopsies of the perforation site and debride the granulation tissue, but the foreign body could not be clearly visualized within the stomach and therefore could not be grasped with forceps for attempted endoscopic removal. The general surgery team arrived to assist with the removal and opted to perform exploratory laparotomy, revealing a 3 cm protruding chicken bone from the anterior surface of the distal gastric wall near the pylorus without any indication of peritonitis, free air, fluid collection, or purulence within the abdomen. As the chicken bone did perforate the gastric tissue, linear gastrostomy was performed to remove the retained foreign body.

**Figure 2:**
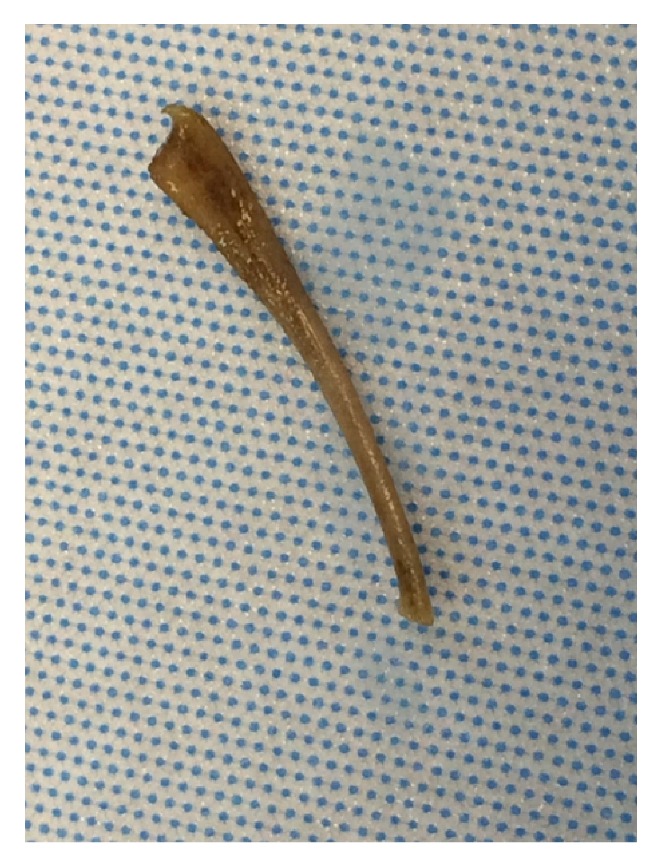
Gross specimen after surgical removal, chicken bone measuring approximately 3 cm in length by 2 mm in width.

The patient had a removal of the chicken bone, with the distal aspect measuring approximately 7 mm in a spatulated form (Figure 2). She had primary sutured closure of the linear gastrostomy followed by normal saline instillation into the abdominal cavity and stomach insufflation to demonstrate no air leak. The abdominal wall was then closed without the need of a peritoneal drainage tube, and the patient was transferred to the recovery room. The patient had an uneventful hospital course with resolution of symptoms and did not require placement of nasogastric tube. She was started on a liquid diet the morning after surgery and slowly advanced to a full diet before discharge to home in stable condition.

## 3. Discussion

Patients with IFB complications such as perforation, abscess formation, or obstruction often present with symptoms of abdominal pain or nausea several days after ingestion [6]. Since most patients do not recall the ingestion, diagnosis is often delayed, with a mean time of 9 days from ingestion to hospital presentation [3]. Therefore, it is crucial to have a high suspicion for IFB in patients who present with typical symptoms, especially in patients with risk factors for IFB. Our patient was an elderly female who ate chicken breast earlier that evening and did not recall the ingestion of the large chicken bone.

IFB is commonly discovered on imaging or even during surgery. Abdominal radiographs usually can not detect IFB, but CT scanning or ultrasonography is very useful for IFB diagnosis [3, 6]. Endoscopic examination can be useful in the diagnosis and management of IFB. If perforation has occurred, surgical correction is recommended, usually involving resection of the affected bowel or local repair [1, 3].

Although surgical intervention remains the mainstay of management of IFB perforations, there have been reports of successful endoscopic management of gastric perforations secondary to IFB in patients who are diagnosed early, prior to the development of complications [4].

Our patient presented only several hours after the chicken bone ingestion and therefore did not develop complications such as abscess formation or peritonitis. However, due to the formation of granulation tissue around the IFB on the intragastric surface, the chicken bone could not be clearly visualized and endoscopic removal was not able to be completed. The patient did ultimately require surgical removal, which was performed via laparotomy due to the preference of the general surgical team. Laparoscopy with or without intraoperative endoscopy is also an option for surgical removal, and there have been reports of successful laparoscopic management of complicated IFB [7].

## 4. Conclusion

If the diagnosis of IFB is made early, invasive surgical interventions can be avoided. Therefore, early recognition of IFB perforation, utilizing clinical suspicion of IFB risk factors and presenting symptoms, is crucial to reduce mortality and complications.
